# FABP3, a Candidate Biomarker of Muscle Strength and Myositis Disease Activity in Patients with Idiopathic Inflammatory Myopathies

**DOI:** 10.3390/biom16101518

**Published:** 2026-10-19

**Authors:** Dorottya Szinay, Katalin Szabó, Tibor Béldi, Viktor Bencs, Sára Csiha, Béla Nagy, Hajnalka Lőrincz, Mariann Harangi, Zoltán Griger, Melinda Nagy-Vincze

**Affiliations:** 1 Division of Clinical Immunology, Department of Internal Medicine, Faculty of Medicine, University of Debrecen, H-4032 Debrecen, Hungary; szinay.dorottya@med.unideb.hu (D.S.); griger.zoltan@med.unideb.hu (Z.G.); 2 Doctoral School of Medical Sciences, University of Debrecen, H-4032 Debrecen, Hungary; 3 Department of Neurology, Faculty of Medicine, University of Debrecen, H-4032 Debrecen, Hungary; 4 Department of Clinical Basics, Faculty of Pharmacy, University of Debrecen, H-4032 Debrecen, Hungary; 5 Department of Laboratory Medicine, Faculty of Medicine, University of Debrecen, H-4032 Debrecen, Hungary; 6 Division of Metabolism, Department of Internal Medicine, Faculty of Medicine, University of Debrecen, H-4032 Debrecen, Hungary; 7 Faculty of Health Sciences, Institute of Health Studies, University of Debrecen, H-4032 Debrecen, Hungary

**Keywords:** polymyositis, dermatomyositis, disease activity marker, fatty acid binding protein 3

## Abstract

**Background:** Identifying novel, reliable blood-based biomarkers is essential to better assess disease activity in idiopathic inflammatory myopathies (IIMs). We aimed to determine serum fatty acid binding protein 3 (FABP3) levels in IIM patients and evaluated their correlations with disease activity, muscle strength, and conventional biomarkers. **Methods:** This cross-sectional study enrolled 80 patients with IIM (49% polymyositis, 51% dermatomyositis) and 47 gender-matched healthy controls. Disease activity was evaluated using the IMACS core set measures, and serum FABP3 levels were determined via ELISA. **Results:** FABP3 levels significantly correlated with creatine kinase (CK; r = 0.42, *p* < 0.001), high-sensitive C-reactive protein (hsCRP; r = 0.35, *p* = 0.002), and physician global disease activity (PhG; r = 0.28, *p* = 0.013), while showing a strong negative correlation with manual muscle testing (MMT; r = −0.39, *p* = 0.0005). Conversely, CK and hsCRP failed to correlate with disease activity or muscle strength. Statistical analysis confirmed that FABP3 is an independent predictor of muscle strength, and correlates with PhG better than CK in patients with chronic myositis. **Conclusions:** FABP3 is a promising biomarker for IIM disease activity, which may enable more effective therapeutic decision-making in clinical practice.

## 1. Introduction

Idiopathic inflammatory myopathies (IIM) represent a heterogeneous group of rare, systemic autoimmune disorders primarily characterized by chronic muscle inflammation and proximal muscle weakness. Beyond skeletal muscle involvement, these conditions frequently affect extra-muscular systems—including the skin, lungs, heart, and gastrointestinal tract—leading to significant morbidity and increased mortality. The classification of IIM has evolved from broad categories into distinct clinicopathological phenotypes based on clinical features, muscle biopsy findings, and myositis-specific antibodies (MSAs). According to the most widely accepted classification systems, the major subsets include dermatomyositis (DM), polymyositis (PM) including immune-mediated necrotizing myopathy (IMNM), antisynthetase syndrome (ASS) and inclusion body myositis (IBM) [1,2,3,4,5,6].

Core set measures (CSMs) to assess IIM disease activity have been established and validated by the International Myositis Assessment and Clinical Studies Group (IMACS) and the Paediatric Rheumatology International Trials Organisation (PRINTO), with provisional endorsement by the American College of Rheumatology and the European League Against Rheumatism [7,8,9]. Core sets include physician and patient global activity, muscle strength, and physical function as well as the most abnormal serum muscle enzyme and extra-muscular global activity. The diagnosis and clinical management of IIM traditionally rely on CSMs and serum creatine kinase (CK) as a primary biomarker for muscle damage. However, a significant subset of patients—particularly those with DM, IBM, or advanced chronic stages of PM—presents with persistently normal or only minimally elevated CK levels despite active disease. Indeed, normal CK levels are common in IIM and do not exclude active muscle involvement [5,10,11]. Due to the expanding knowledge about the pathogenesis of IIM, there is a pressing need for simple blood-based biomarkers of disease activity and organ involvement. Skeletal muscle injury biomarkers, such as aldolase may outperform and complement conventional biomarkers like CK [11]. Consequently, researchers are continuously working on identifying biomarkers, which could facilitate therapeutic decision in cases of muscle weakness [12].

Fatty acid binding protein 3 (FABP3) is a crucial lipid chaperone primarily expressed in cardiac and skeletal muscles, where it facilitates fatty acid uptake and metabolism for energy production. Its significance in muscle diseases spans both its functional role in maintaining muscle homeostasis and its utility as a potential biomarker for muscle injury and pathology [13,14]. FABP3 is integral to the proper functioning of muscle tissue, which derives a significant portion of its energy from fatty acid oxidation. In the context of muscle disorders, altered FABP3 expression has been observed in sarcopenia and ageing. Furthermore, FABP3 overexpression disrupts lipid homeostasis by altering membrane lipid composition, leading to endoplasmic reticulum (ER) stress and inhibited protein synthesis, which subsequently limits muscle recovery [15]. Additionally, plasma concentrations of FABP3 have been shown to positively correlate with disease severity in myasthenia gravis [16].

Studies have indicated elevated FABP3 expression within the skeletal muscle fibres of patients with PM/DM, suggesting its involvement in the pathophysiology of inflammatory myopathies. In IIM, FABP3 production is substantial, characterized by both elevated serum levels and aberrant muscular tissue expression, indicating ongoing muscle damage and inflammation [17]. Furthermore, serum FABP3 levels were significantly higher in patients with active IIM compared to healthy controls or those in remission, particularly during the initial stages of the disease [18]. Previous research has demonstrated that elevated serum FABP3 levels correlate more strongly with the degree of muscle weakness than conventional markers, such as CK or myoglobin [17,18,19,20,21].

Based on the provided literature, further investigation of serum FABP3 levels in IIM patients, their specific clinical subtypes, and healthy controls, alongside its correlation with disease activity, offers several significant scientific and clinical advantages. Evaluating FABP3 across distinct IIM subsets (such as polymyositis and dermatomyositis) will help determine if FABP3 serum concentration is different in specific disease phenotypes. Correlation to clinical disease activity scores will validate its utility as a real-time biomarker and actually help clinicians in the therapeutic decision-making process. Therefore, we aimed to determine FABP3 levels in patients with IIM and to investigate the correlations between FABP3, disease activity, CK levels, and muscle strength. We hypothesized that FABP3 could be a good predictor of muscle strength and physician global disease activity (PhG) in patients with chronic active myositis, which is a critical group in terms of change in therapy.

## 2. Materials and Methods

### 2.1. Study Population

A total of 80 patients with IIM, who were treated and regularly controlled at the Division of Immunology, Department of Internal Medicine, Faculty of Medicine, University of Debrecen, Debrecen, Hungary, participated in the study. To be included, patients had to fulfil the classification criteria of definite or probable inflammatory myopathy based on the EULAR/ACR guidelines [2] and provide written informed consent. Ethical approval for this study was granted by the Institutional Review Board (DE RKEB/IKEB 6215-2022). Epidemiology data, general characteristics of IIM disease—such as clinical symptoms at disease onset, autoantibody profile, comorbidities, and previous immunosuppressive treatment—were collected. Forty-seven gender-matched healthy volunteers were recruited as a control group among the employees of the department. Eligible participants were adults without known autoimmune, inflammatory, metabolic, malignant, renal, hepatic, or cardiovascular diseases and without a history of idiopathic inflammatory myopathy. Individuals with self-reported muscle pain, weakness, known acute infection, chronic inflammatory disorders, diabetes mellitus, active malignancy, or ongoing immunosuppressive treatment were excluded from participation. All healthy participants also provided written informed consent before enrolment.

### 2.2. Disease Activity Measurement

Disease activity was determined using the International Myositis Outcome Assessment Collaborative Study (IMACS) core set measures [21]. Muscle strength was measured with the manual muscle test (MMT) proposed by IMACS. The 8 muscle groups were evaluated as follows: neck flexors, deltoids, biceps, wrist extensors, quadriceps, gluteus medius, gluteus maximus and ankle dorsiflexors. The MMT assesses these muscle groups by testing them bilaterally, except neck flexors. A full score for each muscle group is 10, with the total possible score being 150. The MMT was performed by a trained physician.

Patient and physician global activity was judged from all the information available on the day of visit, including the subject’s appearance, physical examination, laboratory testing and resultant medical therapy. Patients rated their global (overall) disease activity assessment by drawing a vertical mark on a 10 cm visual analogue (VAS) scale. A physician evaluated patient disease activity based on all above-mentioned information, also on a 10 cm VAS scale. Muscle pain was evaluated based on patients’ complaints marking the severity of pain on a 10 cm VAS scale.

### 2.3. Sample Collection and Laboratory Measurements

Routine metabolic laboratory parameters such as hemoglobin A1C (HgbA1c), total cholesterol, triglyceride, high-density lipoprotein cholesterol (HDL-C), LDL-C, apolipoprotein A1 (ApoA1), and apolipoprotein B100 (ApoB100), and inflammatory markers such as high-sensitive C-reactive protein (hsCRP), interleukin 6 (IL-6), creatine kinase (CK), lactate dehydrogenase (LDH), aspartate aminotransferase (AST), alanine aminotransferase (ALT) and cardiac necroenzymes (cardiac troponin T (cTnT)), cardiac troponin I (cTnI), and N-terminal pro-B-type natriuretic peptide (NT-proBNP) were measured from fresh sera with Cobas c600 analyzers (Roche Ltd., Mannheim, Germany) from the same vendor. All venous blood samples were drawn after a 12 h fasting period. The commercially available methods were completed following the manufacturers’ protocols. All laboratory measurements were performed at the Department of Laboratory Medicine, University of Debrecen, Debrecen, Hungary. Two aliquots of serum (each 300 μL) were frozen at −80 °C for the subsequent laboratory measurements. Serum concentrations of FABP3 were detected from undiluted sera by a commercially available enzyme linked immunoassay (ELISA) kit (Cat. No. DY1678; R&D Systems Europe Ltd.; Abington, UK). Serum samples were analyzed according to the manufacturer’s protocol, using the same assay conditions, and measurements were performed in duplicate by the same laboratory expert in the same day to minimize analytical variability. In addition, preliminary laboratory tests were performed to determine the concentration range in serum matrices. According to these measurements, a relatively low FABP3 concentration range was detected; therefore, undiluted samples were used in this assay. Values were expressed in ng/mL.

### 2.4. Statistical Analysis

Statistical analysis was performed with Statistica v.13.5.0.17 software (TIBCO Software Inc., Tulsa, OK, USA). Graphs were made using GraphPad Prism 8.0 (GraphPad Software, San Diego, CA, USA). Normality of variables was checked with the Kolmogorov–Smirnov test. Statistical significance was calculated using the Mann–Whitney U test or unpaired Student’s test among the PM and DM groups. Kruskal–Wallis ANOVA with Bonferroni correction was used for the multiple comparison. Data are presented as mean ± standard deviation (SD) or median (lower–upper quartiles). Pearson correlation analyses were performed to identify the statistical strength between two continuous variables. Skewed variables were logarithmized before the correlation analyses. Backward stepwise multiple regression analyses were performed to identify significant predictor(s) of muscle strength (model 1) and physician global disease activity (model 2). Model 1 included age, sex, myositis disease duration, corticosteroid use, log_10_CRP, log_10_CK, and log_10_FABP3 for muscle strength as a dependent variable (*n* = 78). Model 2 included age, sex, myositis disease duration, corticosteroid use, log_10_CRP, log_10_CK, and log_10_FABP3 for physician global disease activity as a dependent variable (*n* = 78). *p* values ≤ 0.05 were considered statistically significant.

## 3. Results

### 3.1. General Characteristics of Study Population

In our study population, the mean age was 56.2 ± 13.4 years. The majority of our patients were diagnosed with DM (51%), while the minority had PM (49% including IMNM and ASS). Average disease duration at the time of testing was 9 (5–15) years. The major characteristics of the overall study population are presented in Table 1. The study group was characterized dominantly by musculoskeletal involvement; the most prevalent clinical symptoms were muscle weakness (96.25% of patients) and arthralgia (81.25%). Regarding extra-muscular manifestations, skin rashes were frequently presented (70%), while internal organ involvement, such as interstitial lung disease (ILD), dysphagia and myocarditis were less common, observed in 37.5%, 36.3% and 16.3% respectively. Systemic features, such as fever (17.5%) were less frequent. Evaluation of cardiovascular comorbidities within the study group revealed a high prevalence of hypertension (71.25%) and diabetes mellitus (25%). Peripheral arterial disease was only present in 17.50% of cases. Serious acute events were rare: thromboembolic events occurred in 7.50%, and acute myocardial infarction in 3.75% of the population, while no cerebrovascular events were reported. The above-mentioned events happened more than 1 year before the study visit.

### 3.2. Disease Activity and Laboratory Markers

The median global disease activity based on physician opinion was 1 (0–2.5) in the overall myositis cohort (Table 2). Nevertheless, 30% of patients had more than 2 points, which refers to an active disease. Patients evaluated worse disease activity with a median value of 2 (0–4). The medial muscle pain VAS score was 0 (0–2), but 32,5% (*n* = 26) of our patients reported muscle pain during the study visit. The distribution of complaints did not differ in the subgroups (16 patients in the PM group and 10 in the DM group). The median CK level was 90.5 (62–185) IU/l in overall patients. Median PhG was low 1 (0–2.5), but 25% of patients (*n* = 20) had more than 2 points (2,5–7) which refers to mild–moderate disease activity. Furthermore, manual muscle test (MMT) scores varied between 133.5 and 150, with a median value of 145, indicating the presence of mild-to-moderate muscle weakness despite ongoing immunosuppressive therapy. Cardiac necroenzymes including NT-proBNP, cTnT and cTnI were significantly elevated in the PM subgroup compared to the DM group (all *p* < 0.05). Triglyceride, AST and HgbA1c levels were also significantly higher in the muscle dominant subset. Besides hsCRP values were significantly higher in the DM group than in the PM group. The general characteristics and laboratory results of the study subgroups are summarized in Table 2.

### 3.3. FABP3 as a Marker of Muscle Inflammation

**Figure 1:**
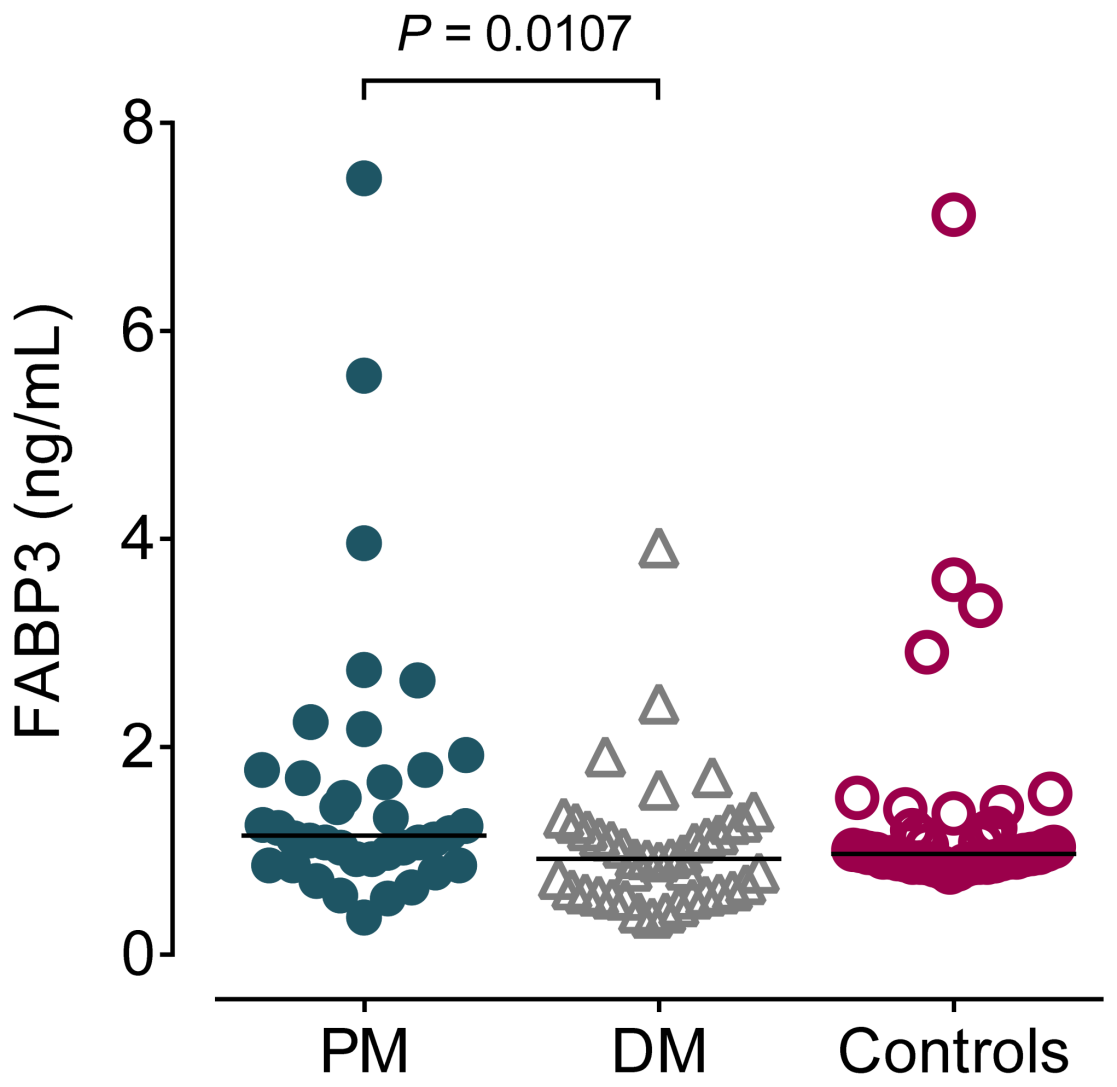
Serum concentration of fatty acid binding protein 3 (FABP3) in patients with polymyositis (PM; 1.15 (0.92–1.78) ng/mL; represented as blue), in patients with dermatomyositis (DM; 0.93 (0.59–1.23) ng/mL; represented as grey) and in controls (0.97 (0.89–1.20) ng/mL; represented as magenta). Solid lines label medians.

The median FABP3 level was 1.08 (0.73–1.38) ng/mL in all IIM patients and 0.97 (0.89–1.20) ng/mL in healthy controls (Table 2). There was no significant difference between patients and controls, only a tendency of higher FABP3 levels when comparing PM patients to healthy people (Table 2 and Figure 1). Significantly higher FABP3 values were measured in the PM subset compared to DM (*p* = 0.0107) (Figure 1).

**Figure 2:**
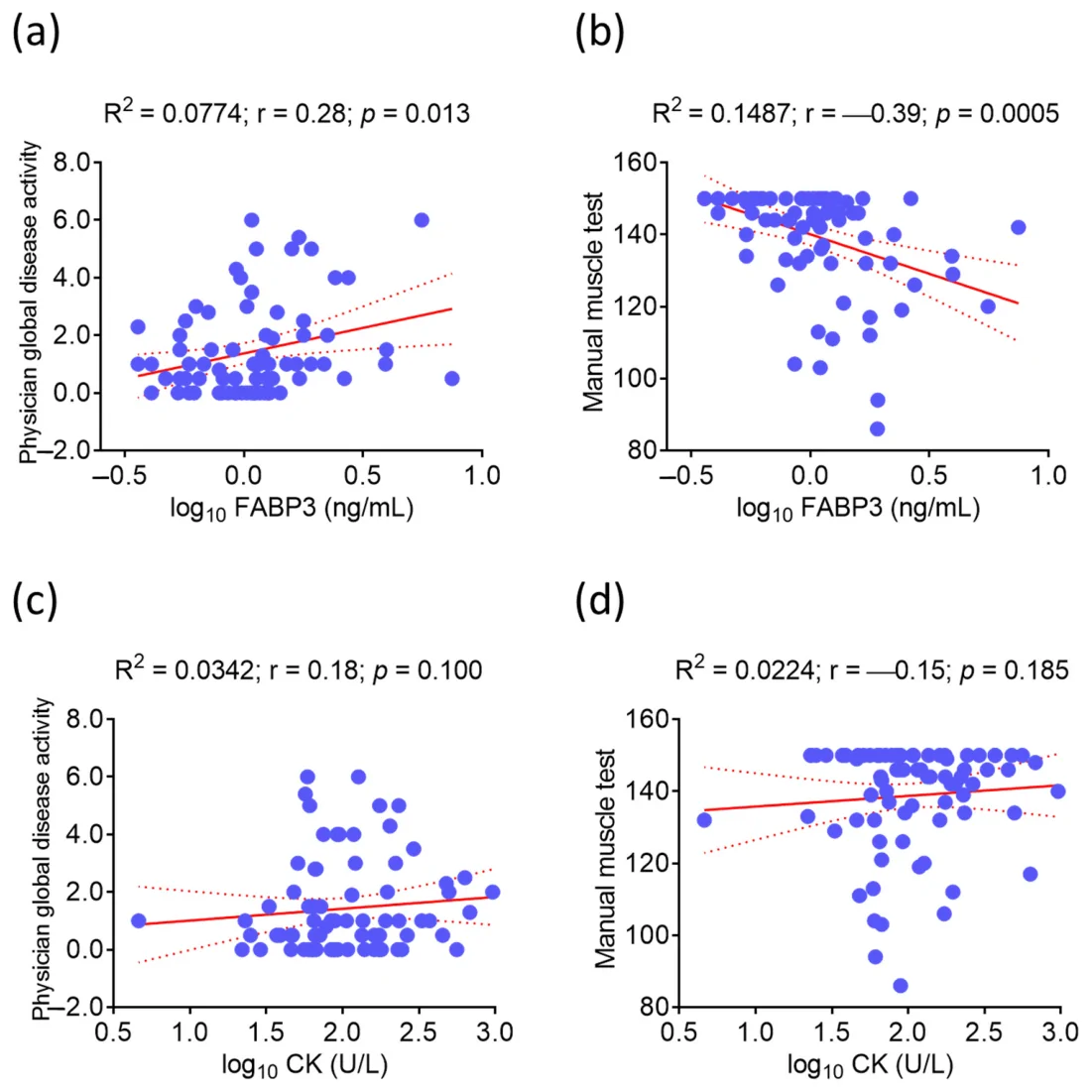
Correlations of physician global activity (**a**) and manual muscle test score (**b**) with serum fatty acid binding protein 3 (FABP3) in overall patients. Correlations of physician global activity (**c**) and manual muscle test score (**d**) with creatine kinase (CK) in overall patients. The solid red lines represent the linear regression trends, while the dotted red lines indicate their corresponding 95% confidence intervals.

Positive correlation was detected between FABP3 and CK (r = 0.42; *p* = 0.00009), PhG (r = 0.28; *p* = 0.013) and hsCRP (r = 0.35, *p* = 0.002), whereas there was a strong negative correlation with MMT (r = −0.39; *p* = 0.0005) (Table 3) in overall IIM patients. FABP3 correlated with the physician global disease activity in patients with activity >2 (r = 0.443; *p* = 0.034), whereas no significant correlation was observed in those with activity ≤2 (r = 0.158; *p* = 0.249). CK showed no correlation with the physician global disease activity at any disease activity level (r = −0.059; *p* = 0.785 and r = −0.078; *p* = 0.568, respectively). Subgroup analysis proved significant positive correlation between physician global disease activity and FABP3 (r= 0.3202; *p* = 0.0427), as well as patient global disease activity and FABP3 (r= 0.3307; *p* = 0.0497) in the muscle dominant subgroup. Meanwhile, a negative correlation was observed between FABP3 and MMT in the DM subset (r = −0.4631; *p* = 0.0026) (Table 3). Among laboratory parameters, CK, cTnT and hsCRP showed positive correlations with FABP3 in the PM subgroup. In the DM patients, cTnT, cTnI, IL-6 and hsCRP correlated positively with FABP3 (Table 3). Meanwhile FABP3 showed correlation with MMT and PhG; CK showed no significant correlation with disease activity, or muscle strength (Figure 2). Correlations of muscle strength, physician global disease activity and patient global disease activity with main laboratory parameters are also summarized in Supplementary Table S1.

Stepwise multiple regression analysis confirmed that FABP3 independently correlated with muscle strength (model 1; *n* = 78; multiple R = 0.480; adjusted R_2_ = 0.154; β_standardized_ = −0.390; *p* < 0.001). Furthermore, among the investigated biomarkers, FABP3 was the strongest predictor of physician global disease activity (model 2; *n* = 78; multiple R = 0.466, adjusted R_2_ = 0.139; β_standardized_ = 0.278; *p* < 0.001). FABP3 showed no significant correlation with any either metabolic or cardiac comorbidities even though we found some differences in cardiac necroenzyme levels between subgroups.

## 4. Discussion

Our study group investigated FABP3 levels and their correlation with disease activity in a large cohort of patients with IIM. To the best of our knowledge, this is the largest study to date investigating the role of FABP3 in patients with IIM. The inclusion of a healthy control cohort in this study serves as a critical methodological baseline and is scientifically justified by several factors. First, establishing a well-defined reference interval within a matched healthy population is mandatory to accurately quantify the degree of serum FABP3 elevation in IIM patients. Given that physiological FABP3 expression can be modulated by non-pathological factors such as lipid metabolism, the healthy cohort helps us ensure that the observed serum fluctuations are genuinely reflective of IIM-specific pathophysiology rather than baseline demographic variances. The patient population consisted predominantly of older individuals, and the recruitment of healthy control subjects of comparable age proved challenging. Nevertheless, we assessed the potential impact of age on serum FABP3 and found no significant correlation between age and this variable within our study population; please see below. Therefore, we believe that the age difference between the groups is unlikely to have influenced the main findings.

Previously published data indicated that while FABP3 shows high specificity (80.6%) in distinguishing between active and inactive myositis, its sensitivity is insufficient for it to be used as a standalone diagnostic marker. Instead, it serves a complementary role, particularly in the absence of significant renal or cardiac impairment, which might otherwise alter its circulating levels [17,18,19,20]. Our results confirm the findings of previous studies conducted on smaller patient cohorts. For instance, Zhang et al. reported that serum FABP3 levels in PM/DM patients (*n* = 24) were significantly increased, with the protein being mainly distributed in slow-twitch muscle fibres. They also identified a negative correlation between serum FABP3 levels and the MMT8 score but found no correlation between creatine kinase (CK) and MMT8. Similarly, Giannini et al. observed that the serum level of FABP was 1.8-fold higher in IIM patients compared to healthy, age-, sex-, and physical-activity-matched volunteers. Furthermore, previous studies reported that FABP3 levels decreased in IIM patients who responded well to treatment (e.g., immunosuppressive therapy) but remained elevated in non-responders [17,18,19,20,21,22,23,24,25,26,27]. In line with these findings, Gupta et al. observed higher serum FABP3 levels in active compared to inactive IIMs and noted a decline in FABP3 levels during follow-up among treatment responders [18].

We observed numerically higher FABP3 levels in the overall IIM population and specifically in PM patients compared to healthy controls. Furthermore, significantly higher FABP3 levels were detected in the PM subgroup than in the DM group. FABP3 values showed a positive correlation with both the physician and the patient global assessment of disease activity. Conversely, a significant negative correlation was found between FABP3 levels and muscle strength evaluated by the manual muscle testing score. Furthermore, the median physician global activity was low (1/10); MMT scores varied still between 133.5 and 150, with a median value of 145, indicating the presence of mild-to-moderate muscle weakness despite ongoing immunosuppressive therapy. A third of patients reported muscle pain (32.5%) and a quarter of patients had disease activity (PhG) above 2. To date there are no validated criteria of clinical remission in IIM. Given the overall low disease activity in the cohort, we additionally examined the associations of FABP3 and CK within the subgroups defined by disease activity scores > 2 and ≤2, based on PRINTO inactivity criteria [28]. FABP3 levels correlated with the PhG in patients with active disease, whereas no significant correlation was observed in the inactive group (activity ≤2). CK showed no correlation with the PhG at any disease activity level. Our results suggest that FABP3 could serve as a promising biomarker, acting as a valuable tool for assessing disease activity in patients with chronic myositis. Taken together, these findings reinforce the potential utility of FABP3 in monitoring IIM disease activity and evaluating treatment efficacy. Nevertheless, further prospective, large-scale cohort studies are warranted to firmly establish the clinical utility and significance of FABP3 as a skeletal muscle biomarker in daily practice. As patients with persisting complaints and decreased muscle strength complicate therapeutic decision-making for clinicians, FABP3 may serve as a potential biomarker to resolve this clinical dilemma.

From a pathophysiological standpoint, the complementary role of FABP3 beside conventional biomarkers like CK in certain patient subsets can be attributed to its molecular properties. FABP3 is a low-molecular-weight cytoplasmic protein (~15 kDa) compared to the significantly larger CK dimer (~80 kDa) [18,24]. Consequently, even minor or early-stage immune-mediated sarcolemma damage and altered membrane permeability can lead to a rapid and substantial leakage of FABP3 into the systemic circulation [26,27]. This rapid kinetic profile explains why serum FABP3 levels serve as a highly sensitive reflection of ongoing microstructural muscle injury and disease activity, even in myositis patients who present with structural chronicity with normal CK levels. Furthermore, accumulating evidence suggests that elevated circulating FABP3 reflects not only active inflammation but also cumulative tissue damage. Chronic metabolic derangements and ischemic-like reperfusion injuries within inflamed muscles promote mitochondrial oxidative stress, lipid peroxidation, and cell apoptosis, all of which drive continuous FABP3 leakage into the bloodstream [26]. This mechanism strongly aligns with recent findings by Giannini et al., who demonstrated that serum FABP3 levels are significantly increased in IIM patients and correlate tightly with total damage extension, severity, and global damage scores evaluated via the International Myositis Assessment and Clinical Studies (IMACS) tool [27]. Our results align with the results of Gupta et al. [18] exactly, that FABP3 levels are indicative of myositis disease activity rather than damage.

As FABP3 is predominantly expressed in cardiomyocytes and skeletal muscle cells, it is not surprising that elevated serum FABP3 levels are closely associated with skeletal muscle pathology. However, underlying cardiac disease could confound elevated FABP3 levels, particularly in middle-aged individuals, especially given the potential for subclinical cardiac involvement. Nevertheless, our patients had no major cardiovascular events in the last 12 months and we found no correlation between FABP3 and cardiovascular comorbidities, suggesting that the elevated levels are primarily related to skeletal muscle disease. In idiopathic inflammatory myopathies (IIMs), elevated serum levels of cardiac troponin T (cTnT) do not exclusively indicate myocardial injury but can frequently originate from regenerating skeletal muscle. Chronic inflammation and subsequent myofiber damage trigger active regeneration, a process that recapitulates embryonic myogenesis and upregulates the transcription of the *TNNT2* gene. This molecular shift results in the re-expression of fetal or embryonic cTnT isoforms within the damaged skeletal muscle tissue. Because these regenerated isoforms share high structural homology with the adult cardiac variant, they cross-react with commercial high-sensitivity immunoassays, producing clinically misleading “false-positive” elevations. In our cohort cTnT also showed positive correlation with disease activity and muscle strength (Supplementary Table S1). Consequently, measuring cardiac troponin I (cTnI)—which maintains absolute tissue specificity and does not undergo re-expression during skeletal repair—is essential to accurately differentiate true myocardial involvement from active skeletal muscle disease [29,30,31]. Consequently, given that FABP3 showed no correlation with metabolic and cardiovascular events or comorbidities within our cohort, we suggest that despite its positive association with cardiac necroenzymes, it functions predominantly as a skeletal muscle biomarker rather than a cardiac one.

## 5. Conclusions

FABP3 correlates well with muscle strength and physician global activity in IIM patients. Therefore, it could serve as a complementary biomarker to guide therapeutic decision-making in patients with a chronic active myositis. Further study of larger myositis patient cohorts with longitudinal data and more diverse activities are required for evaluating clinical relevance.

## Figures and Tables

**Table 1:** General characteristics of study population.

	Characteristics of Study Population (*n* = 80)
Mean age (years ± SD)	56.2 ± 13.4
Duration of disease (years)	9 (5–15)
Gender (female/male)	67.5% (*n* = 54)/32.5% (*n* = 26)
Smoking status (*n*)	17.5% (*n* = 14)
Polymyositis (including ASS and IMNM)	49% (*n* = 39)
Dermatomyositis	51% (*n* = 41)
Myositis specific antibody (MSA) positivity	60% (*n* = 48)
Myositis associated autoantibody (MAA) positivity	43.7% (*n* = 32)
Clinical symptoms	
Muscle weakness	96.25% (*n* = 77)
Skin rashes	70% (*n* = 56)
Interstitial lung disease	37.50% (*n* = 30)
Dysphagia	36.25% (*n* = 29)
Myocarditis	16.25% (*n* = 13)
Arthralgia	81.25% (*n* = 65)
Fever	17.50% (*n* = 14)
Raynaud’s phenomenon	38.75% (*n* = 31)
Cardiovascular comorbidities	
Hypertension	71.25% (*n* = 57)
Diabetes mellitus	25% (*n* = 20)
Thromboembolic events	7.50% (*n* = 6)
Peripheral arterial diseases	17.50% (*n* = 14)
Cerebrovascular events	0% (*n* = 0)
Acute myocardial infarction	3.75% (*n* = 3)

**Table 2:** Anthropometric data, disease activity and laboratory parameters of enrolled subjects.

	All IIM	PM	DM	Controls
Anthropometric parameters				
Number of patients, M/F (*n*)	80 (26/54)	39 (14/25)	41 (12/29)	47 (16/31)
Age (years)	56.2 ±13.4	58.9 ± 12.8 a	53.5 ± 13.5 b	42.9 ± 8.6
Disease activity				
Patient global activity	2 (0–4)	2 (0–3)	2 (0–4)	n.a.
Physician global activity	1 (0–2.5)	1 (0–2)	1 (0–2.5)	n.a.
Manual muscle test score	145 (133.5–150)	143 (129–150)	146 (137–150)	n.a.
Muscle pain VAS score	0 (0–2)	0 (0–2)	0 (0–0)	n.a.
Laboratory parameters				
CK (U/L)	90.5 (62–185)	93.0 (64.0–222.0)	89.0 (60.0–175.0)	n.a.
LDH (U/L)	217.1 ± 60.4	240.8 ± 79.0	216.2 ± 41.3	n.a.
NT-proBNP (ng/L)	73.2 (24.5–388.9)	114.7 (44.8–388.9) *	43.6 (24.5–102.3)	n.a.
cTnT (ng/L)	10.4 (5.2–42.1)	12.1 (7.1–42.1)	7.7 (5.2–14.8)	n.a.
cTnI (ng/L)	3.2 (1.7–7.6)	4.4 (2.4–7.6) *	2.4 (1.7–3.9)	n.a.
Interleukin-6 (pg/mL)	2.75 (1.5–9.5)	3.36 (1.57–9.50)	2.08 (1.50–6.96)	n.a.
Total cholesterol (mmol/L)	5.78 ± 1.42	5.89 ± 1.56 a	5.55 ± 1.27	5.03 ± 0.73
HDL-C (mmol/L)	1.56 ± 0.6	1.58 ± 0.62	1.56 ± 0.55	1.48 ± 0.44
LDL-C (mmol/L)	3.44 ± 1.18	3.45 ± 1.28	3.42 ± 1.10	2.95 ± 0.53
ApoA1 (g/L)	1.65 ± 0.36	1.66 ± 0.36	1.64 ± 0.36	1.59 ± 0.30
ApoB100 (g/L)	1.11 ± 0.31	1.16 ± 0.33 a	1.06 ± 0.28	0.94 ± 0.17
Triglyceride (mmol/L)	1.7 (1.2–2.4)	1.76 (1.41–2.63) *a	1.47 (0.96–1.99) b	1.1 (0.9–1.6)
hsCRP (mg/L)	3.655 (1.2–6.6)	3.0 (1.2–6.6) *	3.9 (2.0–5.9) b	1.4 (0.6–2.5)
Hb_A1C_ (%)	6.05 ± 0.88	6.2 ± 1.0 *a	5.9 ± 0.8 b	5.1 ± 0.3
AST (U/L)	25.0 (17.0–27.0)	24.0 (18.0–31.0)	19.0 (17.0–23.0)	19.0 (17.0–24.0)
ALT (U/L)	20.0 (14.0–32.0)	20.0 (15.0–32.0)	19.0 (14.0–24.0)	17.0 (12.5–23.5)
GGT (U/L)	25.5 (17.0–71.0)	26.0 (18.0–71.0)	21.0 (17.0–38.0)	19.0 (16.0–28.5)
Myoglobin (μg/L)	33.4 (21.8–52.9)	34.2 (23.6–46.4)	25.8 (21.0–56.2)	n.a.

Abbreviations: ALT, alanine aminotransferase; ApoA1, apolipoprotein A1; ApoB100, apolipoprotein B100; AST, aspartate aminotransferase; CK, creatine kinase; cTnT, cardiac troponin T; cTNI, cardiac troponin I; DM, dermatomyositis; FABP3, fatty acid binding protein 3; GGT, gamma-glutamyl transferase; HbA1C, hemoglobin A1C; HDL-C, high-density lipoprotein cholesterol; hsCRP, high-sensitive C-reactive protein; IIM, idiopathic inflammatory myopathies; LDH, lactate dehydrogenase; LDL-C, low-density lipoprotein cholesterol; n.a., not available; PM, polymyositis. Values are presented as mean ± SD or median (interquartile ranges). * indicates *p* < 0.05 between PM vs. DM; a indicates *p* < 0.05 between PM vs. controls; b indicates DM vs. controls. Only *p* values which passed the Bonferroni correction are presented (*p* < 0.002).

**Table 3:** The correlations of fatty acid binding protein 3 (FABP3) with laboratory and disease activity parameters in all study patients with idiopathic inflammatory myositis, as well as in the polymyositis and dermatomyositis subsets.

Variables	All IIM			PM			DM		
	R^2^	r	*p*	R^2^	r	*p*	R^2^	r	*p*
log_10_ CK (U/L)	0.0936	0.4200	<0.0001	0.1641	0.4051	0.0116	0.0128	0.1131	0.4871
log_10_ NT-proBNP (ng/L)	0.0292	0.1709	0.1347	0.0001	0.0113	0.9464	0.0323	0.1796	0.2674
log_10_ cTnT (ng/L)	0.4754	0.6895	<0.0001	0.4677	0.6839	<0.0001	0.3816	0.6177	<0.0001
log_10_ cTnI (ng/L)	0.1612	0.4015	0.0003	0.0296	0.1720	0.3017	0.2709	0.5205	0.0006
Interleukin-6 (pg/mL)	0.1677	0.4095	0.0002	0.0881	0.2969	0.0703	0.2577	0.5077	0.0008
log_10_ hsCRP (mg/L)	0.1237	0.3518	0.0016	0.1367	0.3698	0.0223	0.1764	0.4199	0.0070
Physician global activity	0.0774	0.2800	0.0130	0.1090	0.3302	0.0429	0.0512	0.2262	0.1605
Patient global activity	0.0855	0.2924	0.0094	0.1028	0.3307	0.0497	0.0470	0.2167	0.1793
Manual muscle test score	0.1487	−0.3900	0.0005	0.0826	−0.2874	0.0802	0.2145	−0.4631	0.0026

Abbreviations: CK, creatine kinase; cTnT cardiac troponin T; cTnI cardiac troponin I; DM, dermatomyositis; hsCRP, high-sensitive C-reactive protein; IIM, idiopathic inflammatory myopathies; PM, polymyositis. Skewed variables were logarithmized before Pearson correlation analyses.

## Data Availability

The data presented in this study are available on request from the corresponding author due to privacy reasons.

## Supplementary Materials

The following supporting information can be downloaded at https://www.mdpi.com/article/10.3390/biom16101518/s1, Supplementary Table S1: Correlations of muscle strength, physician global disease activity and patients’ global disease activity with laboratory parameters in all study patients with idiopathic inflammatory myositis.

## Abbreviations

The following abbreviations are used in this manuscript:

ACR	American College of Rheumatology
ALT	alanine aminotransferase
ApoA1	apolipoprotein A1
ApoB100	apolipoprotein B100
ASS	antisynthetase syndrome
AST	aspartate aminotransferase
CK	creatine kinase
CTnI	cardiac troponin I
CTnT	cardiac troponin T
DM	dermatomyositis
ELISA	enzyme linked immunoassay
ER	endoplasmic reticulum
EULAR	European Alliance of Associations for Rheumatology
FABP3	fatty acid binding protein 3
GGT	glutamyl transpeptidase
HDL-C	high-density lipoprotein cholesterol
HgbA1c	hemoglobin A1C
hs CRP	high-sensitive C-reactive protein
kDa	kilodalton
IBM	inclusion body myositis
IIM	idiopathic inflammatory myopathies
ILD	interstitial lung disease
IL-6	interleukin-6
IMACS	International Myositis Outcome Assessment Collaborative Study
IMNM	immune-mediated necrotizing myopathy
LDH	lactate dehydrogenase
LDL-C	low-density lipoprotein-cholesterol
MAA	myositis associated autoantibody
MMT	manual muscle test
MSA	myositis specific autoantibody
NT-proBNP	N-terminal pro-B-type natriuretic peptide
PhG	physician global disease activity
PM	polymyositis
VAS	visual analogue scales
