# Supplementary material for: FABP3, a Candidate Biomarker of Muscle Strength and Myositis Disease Activity in Patients with Idiopathic Inflammatory Myopathies
Source: Biomolecules. 2026 Oct 19;16(10):1518. doi: 10.3390/biom16101518 (PMC13702643; doi:10.3390/biom16101518)
Supplement: Supplementary file 1 [file biomolecules-16-01518-s001.zip › biomolecules-4538887-supplementary.pdf]

Supplementary Table S1. Correlations of muscle strength, physician global disease activity and patients' global disease activity with laboratory parameters in all study patients with idiopathic inflammatory myositis.

Variables	Muscle strength	Physician global disease activity	Patient global disease activity
log_{10} FABP3 (ng/mL)	R^2=0.1487;<br>r=0.-3900;<br>p=0.0005	R^2=0.0774;<br>r=0.2800;<br>p=0.0130	R^2=0.0855;<br>r=0.2924;<br>p=0.0094
log_{10} CK (U/L)	R^2=0.0224;<br>r=-0.1500;<br>p=0.1850	R^2=0.0342;<br>r=0.1800;<br>p=0.1004	R^2=0.0246;<br>r=0.1569;<br>p=0.1644
log_{10} myoglobin (μg/L)	R^2=0.0209;<br>r=-0.1447;<br>p=0.3161	R^2=0.0034;<br>r=0.0581;<br>p=0.6884	R^2=0.0155;<br>r=0.1243;<br>p=0.3897
log_{10} cTnT (ng/L)	R^2=0.1458;<br>r=-0.3818;<br>p=0.0005	R^2=0.0626;<br>r=0.2501;<br>p=0.0253	R^2=0.0514;<br>r=0.2268;<br>p=0.0431

Abbreviations: CK, creatine kinase; cTnT, cardiac troponin T; cTNI, cardiac troponin I; FABP3, fatty acid binding protein 3. Skewed variables were logarithmized before Pearson correlation analyses.
